# Cerebrovascular ischemia following ultrasound-guided foam sclerotherapy

**DOI:** 10.1590/1677-5449.004418

**Published:** 2018

**Authors:** Agamenon Hülse Bittencourt, Daniela Vianna Dallanora, Nelson Rafael Bacega, Vinicius Saul Cembranel

**Affiliations:** 1 Universidade do Oeste de Santa Catarina – UNOESC, Joaçaba, SC, Brasil.; 2 Hospital Santa Maria – HSM, Serviço de Cirurgia Vascular, Videira, SC, Brasil.

**Keywords:** varicose veins, sclerotherapy, cerebral ischemia, varizes, escleroterapia, isquemia cerebral

## Abstract

Cerebral ischemia is a very rare and harmful complication of ultrasound-guided foam sclerotherapy for treatment of varicose veins. This case describes a documented cerebrovascular ischemia in Broca’s area following ultrasound-guided foam sclerotherapy. Less than one hour after intravenous injection of 10 ml of sclerosing foam, an otherwise healthy woman experienced aphasia without any other signs of neurological changes. When she arrived home, a complete inability to talk was observed. The event was misdiagnosed by another doctor as an allergic reaction. Next morning she came to the office to report the allergic reaction, where an appropriate diagnosis was made. She recovered just two days after the injection, but signs of recent cerebral ischemia were seen in Broca’s area on magnetic resonance and transesophageal bubble study echocardiogram revealed a patent foramen ovale. Although rare, we must make great effort to prevent these events instead of treating them.

## Resumo

A isquemia cerebral é uma complicação muito rara e perigosa da escleroterapia com espuma guiada por ultrassom no tratamento de varizes. Este caso descreve uma isquemia cerebral da área de Broca após escleroterapia com espuma guiada por ultrassom. Menos de uma hora após injeção de 10 mL de espuma, uma até então saudável mulher apresentou afasia, sem quaisquer outros sinais neurológicos. No caminho para casa, uma completa incapacidade de fala foi observada. Levada ao hospital mais próximo, foi diagnosticada com reação alérgica. Na manhã seguinte, compareceu ao consultório para relatar a reação alérgica, quando um correto diagnóstico foi feito. Ela ficou recuperada dois dias depois do procedimento, mas uma ressonância magnética mostrou sinais de isquemia recente da área de Broca e um ecocardiograma transesofágico mostrou um forame oval patente. Embora raros, devemos concentrar nossos esforços em prevenir tais eventos para que não precisem de tratamento.

## INTRODUCTION

 Considering that varicose veins are a very prevalent disease and that advances in vascular medicine have enabled us to perform less invasive procedures, chemical ablation with ultrasound-guided foam sclerotherapy (UGFS) has become more and more common, but so have its complications. 

 Foam sclerotherapy can cause drug and/or gas-related complications of a generalized or localized nature. Significant complications include anaphylactic/anaphylactoid reactions (very rare), deep vein thrombosis (1-3%), cerebrovascular accidents (CVA) (<0.01%), superficial venous thrombosis (4.4%), tissue necrosis (of variable frequency), edema (0.5%), and nerve damage (0.2%). Cosmetic complications include telangiectatic matting (15-24%) and pigmentation (10-30%). Patent foramen ovale (PFO) and other cardio-pulmonary right-to-left shunts seem to play a role in systemic gas-related complications. 1

## CASE DESCRIPTION

**Figure 1:**
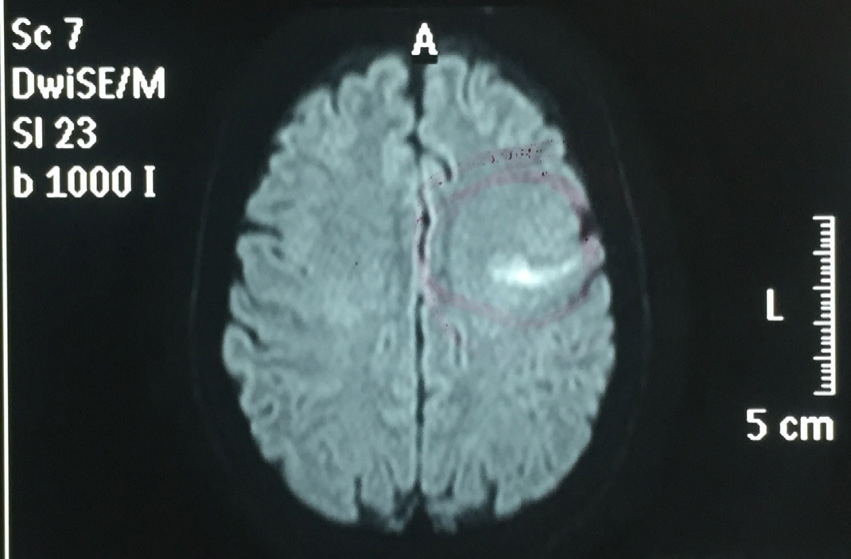
Magnetic resonance showing area of recent ischemia in Broca’s area, where A indicates anterior position of brain’s patient.

**Figure 2:**
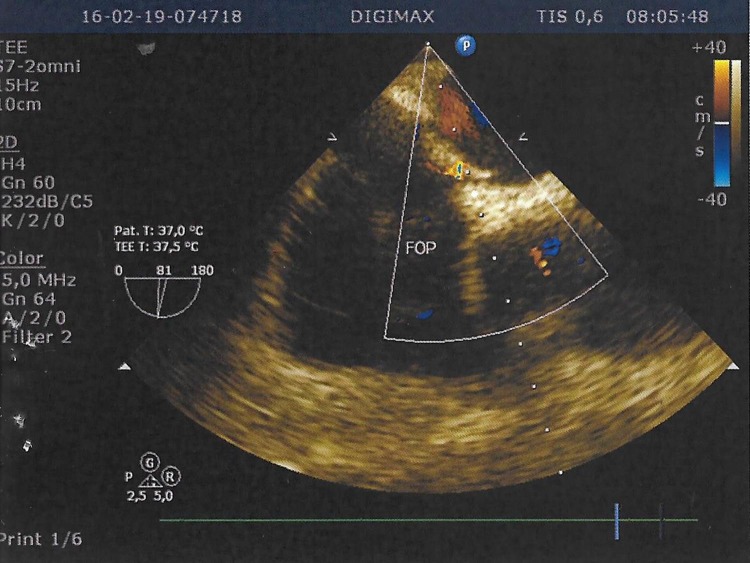
Transesophageal bubble study echocardiogram showing a patent foramen ovale (FOP).

 A 64-year-old woman presented with large varicose veins, CEAP C2s, in the left lower limb. The patient had never been treated before because she was afraid of surgery. She had no history of migraine headaches or cardiac diseases. She had never smoked cigarettes, never had high blood pressure, and never been overweight. Her only medication was 20 mg sinvastatin once a day, and her last lipid profile, and all blood tests were normal. Except for the large varicose veins in her left lower limb, physical examination was normal, including normal peripheral pulses and absence of bruits. The patient was treated in the Trendelenburg position with a total of 10 ml of 3% polidocanol foam via direct punctures, 5 mL into an 8 mm diameter great saphenous vein and 5 mL into large collaterals in the leg. Foam was prepared with a 1:4 ratio of liquid to room air, using the Tessari technique involving 40 passes of agitation through a three-way stopcock using one 5 mL syringe and one 3 mL syringe. With ultrasound guidance, foam was injected immediately after each of three preparations, 5 mL, 2.5 mL, and 2.5 mL. No air boluses occurred. Ultrasound scanning showed no foam in the deep venous system. The patient remained lying down for 10 minutes after the injections, before being discharged wearing compression stockings. Less than 1 hour after leaving hospital, impairment of speech capacity was observed. She encountered difficulties when she tried to talk, with incomplete and incomprehensible words. No other alterations were noted. She was taken to another hospital, where the clinical presentation was misdiagnosed as an allergic reaction. One gram of hydrocortisone IV was infused and 20 mg prednisone was prescribed per day for 5 days. The next morning she came to the office to report the allergic reaction. After detailed history taking and physical examination, including the Wells DVT clinical model, the only alteration detected was aphasia. This was Broca’s aphasia, identified by loss of speech and writing capabilities, with no impairment of comprehension. A neurological examination found no signs or symptoms of hemiparesis or hemiplegia. Cerebrovascular ischemia was suggested, and the patient’s daughter became fairly angry about the diagnosis of ischemia, which she considered exaggerated. Both mother and daughter left the clinic and refused a neurological consultation. Two days later, the patient came to the office, reporting that she was almost recovered and reaffirming her belief that she had suffered an allergic reaction. After an appropriate explanation, 1 week later, a normal transthoracic echocardiogram was obtained and magnetic resonance showed a recent cerebrovascular ischemia in Broca’s area ( Figure 1 ). A transesophageal bubble study echocardiogram performed a few days later revealed a patent foramen ovale ( Figure 2 ). The patient’s daughter’s became very upset again, and, despite a complete recovery by the patient, the doctor-patient relationship broke down almost completely. It wasn’t possible to continue investigations. More explanation and discussion followed, in which the intention to publish this complication was decisive to achieving greater comprehension about what had occurred. Both the patient and her daughter gave permission for publication in writing. 

## DISCUSSION

 Neurological side effects following sclerotherapy are a rare occurrence, but cerebrovascular accidents associated with the use of sclerotherapy have been clearly documented. A recent meta-analysis of the frequency of serious neurological events in patients treated with foam sclerotherapy has reported rates as high as 0.9% with 12 radiologically confirmed CVAs and 9 reported transient ischemic attacks. Many, though not all, of these patients were found to have a PFO. 2

 Current guidelines call for administration of no more than 10 cc of foam and recommend room air (Level 1A recommendation) or a mixture of carbon dioxide and oxygen (Level 2B recommendation). 3 ^,^ 4

 Given the high estimated prevalence of patent foramen ovale and atrial septal defect, from 34.3% during the first three decades of life to 25.4% during the 4th through 8th decades, and to 20.2% during the 9th and 10th decades, there appears to be a very large population exposed to risk of arterial foam embolization. 5

 The Tessari technique is inherently imprecise at creating a standardized bubble mixture size in the laboratory, let alone the real world where clinicians may vary angles in a three way stopcock, the ratio of gas to liquid, and timing of administration pending intravenous access. The Double Syringe System (DSS) technique, which eliminates the third channel and reduces variability introduced by the stopcock angle may further improve consistency. 6

 The role of compounded versus branded preparations of polidocanol and sotradecol and their potential role in central nervous system effects has not been elucidated. Endovenous polidocanol microfoam (PEM, BTG, London, UK) is a commercially available preparation which combines physiologic gas with 0.5% and 1.0% polidocanol in a consistent, predictable fashion. In studies reported to date, no severe cerebrovascular events or migraine headaches have been reported. 7

 Transesophageal echocardiography (TEE) and transesophageal bubble study echocardiography (TBSE) have proven to be important methods of PFO diagnosis. The proximity of the esophagus to the heart and great vessels offers an excellent ultrasonic view, providing accurate information when compared to transthoracic echocardiography (TTE). 8 ^,^ 9

 Reeves et al. say that the utility of TEE can be classified according to general indications and specific indications (intraoperative and other procedural guidance). The indications for TEE include evaluation of the cardiac and aortic structures, in situations in which findings would change management of the patient and in cases in which an accurate diagnosis isn’t possible with TTE (detailed evaluation of the abnormalities in structures such as the aorta and left atrium, evaluation of prosthetic heart valves, evaluation of valve masses, and other uses). 8 ^,^ 10

 Since TEE is more invasive than TTE, its safety has been documented in multiple studies and the risks and complications related to TEE have recently been reviewed. Overall complications related to TEE vary from 0.18 to 2.8%, while mortality varies from <0.01 to 0.02% when it is used for diagnostic proposes. 8 ^,^ 11 ^-^ 13

 As computed tomography angiography (CTA) technology has advanced, clinicians have discovered that PFO is a frequent finding in routine coronary CTA, and interest in this issue has grown. The high spatial and temporal resolution provided by advances in CTA technology have enabled easy evaluation of the interatrial septum, offering a new perspective, from which coronary CTA constitutes a more practical and efficient alternative to TEE for PFO diagnosis. 14 ^-^ 17

 Thus, if TEE complications are more prevalent than neurological complications related to UGFS and more studies are needed to elucidate the role of CTA in PFO diagnosis for prevention of neurological complications in UGFS, we must concentrate all efforts on investigating other aspects. 

 Taking a global view of this case and exploring all possible differential diagnoses, embolization via the foramen ovale, embolization via the carotid territory, and embolization related to deep venous thrombosis must all be considered. 

 Without imaging exams, just working in the field of theoretical sources of embolus, there is no possible way to undoubtedly rule out any of these three hypotheses. However, some considerations can be raised. 

 The physical examination of the lower limbs was normal. This cannot per se rule out deep venous thrombosis, but in combination with other features of Wells’ clinical model it can be considered a less probable cause of this cerebral embolization. 18

 Carotid arteries as source of this embolization can almost be ruled out, considering the very low probability of an event that coincides in time, occurring on the same day and at the same time as UGFS. 

 With regard to embolization via the foramen ovale itself, only a previous bubble study TEE could have shown a thrombus in the foramen ovale. As discussed in this case, it does not seem feasible to order a TEE prior to UGFS for all patients. 

 Continued investigations of patient selection, the potential roles of the product, gas, volume, and techniques in order to identify optimal approaches and products may further define the neurological safety of foam sclerotherapy. 
